# Modelling of Usual Nutrient Intakes: Potential Impact of the Choices Programme on Nutrient Intakes in Young Dutch Adults

**DOI:** 10.1371/journal.pone.0072378

**Published:** 2013-08-28

**Authors:** Annet J. C. Roodenburg, Adriana J. van Ballegooijen, Mariska Dötsch-Klerk, Hilko van der Voet, Jacob C. Seidell

**Affiliations:** 1 Unilever Research & Development Vlaardingen, Unilever, Vlaardingen, The Netherlands; 2 Department of Health Sciences, VU University Amsterdam, Amsterdam, The Netherlands; 3 Sector Food, HAS University of Applied Sciences, Den Bosch, The Netherlands; 4 Department of Human Nutrition, Wageningen UR, Wageningen, The Netherlands; 5 Biometris, Wageningen UR, Wageningen, The Netherlands; National Institute of Agronomic Research, France

## Abstract

**Introduction:**

The Choices Programme is an internationally applicable nutrient profiling system with nutrition criteria for trans fatty acids (TFA), saturated fatty acids, sodium, added sugar and for some product groups energy and fibre. These criteria determine whether foods are eligible to carry a “healthier option” stamp. In this paper a nutrient intake modelling method is described to evaluate these nutritional criteria by investigating the potential effect on nutrient intakes.

**Methods:**

Data were combined from the 2003 Dutch food consumption survey in young adults (aged 19–30) and the Dutch food composition table into the Monte Carlo Risk Assessment model. Three scenarios were calculated: the “actual intakes” (scenario 1) were compared to scenario 2, where all foods that did not comply were replaced by similar foods that did comply with the Choices criteria. Scenario 3 was the same as scenario 2 adjusted for the difference in energy density between the original and replacement food. Additional scenarios were calculated where snacks were not or partially replaced and stratified analyses for gender, age, Body Mass Index (BMI) and education.

**Results:**

Calculated intake distributions showed that median energy intake was reduced by 16% by replacing normally consumed foods with Choices compliant foods. Intakes of nutrients with a maximal intake limit were also reduced (ranging from −23% for sodium and −62% for TFA). Effects on intakes of beneficial nutrients varied from an unintentional reduction in fat soluble vitamin intakes (−15 to −28%) to an increase of 28% for fibre and 17% calcium. Stratified analyses in this homogeneous study population showed only small differences across gender, age, BMI and education.

**Conclusions:**

This intake modelling method showed that with consumption of Choices compliant foods, nutrient intakes shift towards population intake goals for the nutrients for which nutrition criteria were defined, while effects on beneficial nutrients were diverse.

## Introduction

Overconsumption of energy dense, nutrient poor diets is one of the largest problems in modern society, resulting in an increasing prevalence of chronic, non-communicable diseases in many countries [1]. In the Dutch population [2], [3], but also in other populations, adherence to dietary guidelines is low [4].

Following a diet consistent with dietary recommendations [1] may reduce the risk of chronic diseases. Therefore in 2004, WHO launched the Global Strategy on Diet, Physical Activity and Health [5] in which one of the recommendations to the private sector was to limit the levels of TFA, SAFA, salt and free sugars in existing products in order to contribute to reducing the burden of chronic diseases [1].

This discrepancy between dietary recommendations and actual intakes forms the basis of various initiatives in defining targets for food reformulation [6], [7]. A nutrient profiling system, which is a systematic method for categorising foods according to their nutritional quality, is often used as basis for food reformulation. Over the years, different systems have been launched, each with another approach and purpose [6]–[9]. It is difficult to compare these systems as there is no gold standard for comparison, however, validation of nutrient profiling systems is of paramount importance [10].

The Choices Programme is an internationally applicable nutrient profiling system with criteria that determine whether foods are eligible to carry a “healthier option” stamp [9]. The aims of the Choices Programme are to stimulate product reformulation and to help consumers by making healthier choices easier to identify. To develop the nutrient profiles for the Choices Programme, the generic criteria for energy and the key nutrients (TFA, SAFA, sodium, added sugar and fibre) were based on international nutrient intake recommendations for daily diets [11]. There is increasing recognition and appreciation of the stamp by consumers [12] and since the launch of the initiative in 2006 it has driven food reformulation into a more healthy direction [13]. The ultimate goal is to meet the recommendations for population intakes. It is hypothesized that if consumers choose food products that comply with these criteria, the calculated daily intake of the key nutrients should improve in the direction of the nutrient intake recommendations. We evaluated the potential impact of Choices on a broad range of nutrient intakes in a Dutch population of young adults aged 19–30 years using a Monte Carlo Risk Assessment (MCRA) model [14].

This paper builds on a short communication published earlier [15]. Now we give a more in depth description of the methodology used; we expanded the analyses to a broader set of nutrients (including vitamins and minerals); we studied the effects of replacing snacks in more detail; we also evaluated whether effects differed between gender, BMI and level of education, to illustrate the possibilities of the intake modelling methodology used.

### Background on the Modelling of Usual Nutrient Intakes

Usual nutrient intake is defined as the long-run average of daily nutrient intakes aggregated over all foods consumed. At the individual level, usual intake is generally unknown because only a few days are observed. However, the distribution of usual intakes in a population can be estimated from short-term measurements such as repeated 24-h recalls by statistical methods [16], [17].

**Figure 1:**
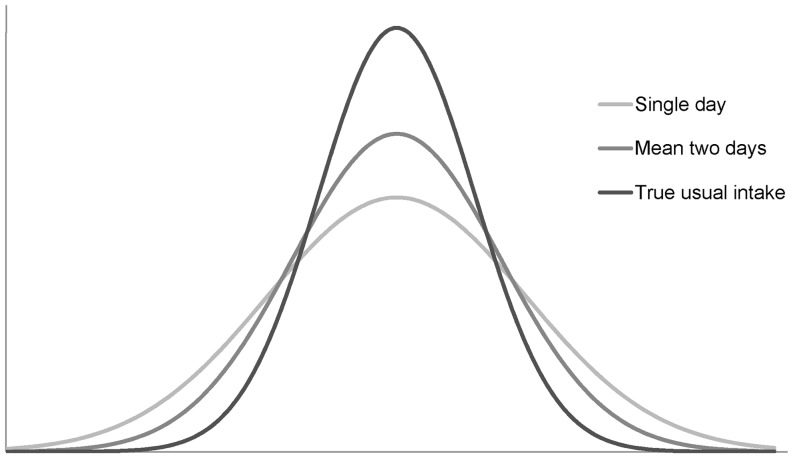
Nutrient intake distributions. A simple estimate of usual intake is to calculate mean intake over the available two days. However, this measure still includes appreciable random error, resulting in a too broad distribution: Both the low-end tail and the high-end tail of the “Mean for two days” distribution over-estimate the frequency of true low and high intakes.

In the current survey, the available consumption data were two 24-h dietary recalls for 750 persons [2]. First, multiplying daily food consumption with nutrient levels per food gives the daily intakes per food on the two days per person for which consumption data are available. Second, aggregating these intakes over all foods gives the total nutrient intake per person per day. A simple estimate of usual intake is to calculate mean intake over the available two days. However, these so-called observed individual means (OIMs) measure the actual usual intake with appreciable random error, and as a consequence the distribution of OIMs is too broad: both the low-end tail and the high-end tail of the OIM distribution over-estimate the frequency of true low and high intakes (see Figure 1, green curve, mean two days).

For nutrients, estimating usual intake is done as follows: first transform the data to a scale where the distribution is approximately normal. This is needed because the error-correction model is based on this assumption. A simple transformation often used for this purpose is the Box-Cox transformation [18]. The second step is fitting a variance components model to the transformed intakes to distinguish between between-person and within-person variation: each intake for a person-day is modelled as 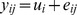, where for individual *i* the term *u_i_* represents the usual intake and *e_ij_* is the intake on day *j* minus the usual intake. By fitting the model, the variance of *u_i_ (Var_u_)* (between-person variance) and the variance of *e_ij_ (Var_e_)* (within-person variance) are estimated. The total variance is the sum of the two variance components: 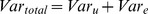. For the purpose of assessing the sufficiency of nutrient intakes day-to-day variations (within person) are irrelevant because health status is generally reflecting long-term rather than short-term intake. Therefore at this stage, to represent the usual intake distribution, the within-person variation is no longer needed. We can continue with only the between-person variation around the mean. Finally, the estimated distribution of *u_i_* is back-transformed to the original scale. An indicator to characterise the difference between the distribution of single-day intakes and the estimated distribution of usual intakes is the so-called “shrinkage factor” which is defined as the ratio of standard deviations for between-person and total variation at the transformed scale (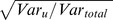).

In this study, we applied the BetaBinomial-Normal (BBN) model that has been developed to quantify the risk of exposure to chemicals from the diet [16], [19], [20]. This model can be applied for nutrients as well as foods, where many of the intakes -at day level- are often zero. In which case, in addition to the normal distribution fitted to the transformed intake amounts, the BetaBinomial part of the model estimates the frequency of consumption of non-daily used foods. However, the BetaBinomial part is not needed for nutrients that are consumed daily as in the current study.

### Cut-point Method

Not only intakes vary between persons, but also nutrient requirements. If assumptions are satisfied, the Average Nutrient Requirement (ANR) cut-point method [21], [22] provides a way to estimate the prevalence of inadequate nutrient intake in a population: by calculating the proportion of estimated usual intake distribution below the ANR. Note that the Average Nutrient Requirement (ANR) and not the level recommended for most individuals (Recommended Daily Allowance, RDA) should be used as a point of comparison, as is fully explained in reference [22].

Assumptions are that intakes are accurately measured and corrected for within-person error, that intakes and requirements are uncorrelated, that the requirements distribution is symmetrical, that the actual prevalence is not very high or very low, and that the variability of requirements in the population can be assumed to be much smaller than the variability in usual intakes.

The population nutrient intake goals of FAO/WHO are also meant to represent average rather than individual values in a healthy population [23], therefore the cut-point method using these limits is appropriate to estimate the proportion of inadequate (too high) intakes of saturated fatty acids, transfatty acids, sodium and added (free) sugar.

## Methods

For a selection of nutrients (carbohydrates, protein, fat, SAFA, TFA, sodium, total sugar, fibre, polyunsaturated fatty acids (PUFA), monounsaturated fatty acids (MUFA), calcium, potassium, iron, folic acid, vitamin A, B_1_, B_2_ B_6_, B_12_, C, D, E) and energy, three scenarios were calculated and compared: In scenario 1 data from the Dutch National Food Consumption Survey 2003 [2] were used to estimate the usual intake distribution (see Background). In this paper we identify this basic estimate of the usual intake distribution as the “Actual intake”. This was compared to scenario 2, which was the same as scenario 1, except that all foods that did not comply with the Choices criteria were replaced –where possible– by similar foods that did comply with the Choices criteria. Scenario 3 was the same as scenario 2, but corrected for the difference in energy density between the original and the replacement food. Two additional scenarios (4 and 5) were calculated for a selection of nutrients: In scenarios 4 and 5, snacks were either not replaced or partially replaced, to estimate the contribution of the different types of snacks to the nutrient intakes.

We applied the BetaBinomial-Normal (BBN) model that was developed to quantify the risk of exposure to chemicals from the diet [16], [19], [20]. The BBN model is available in the Monte Carlo Risk Assessment (MCRA) program [14]. Inputs for this model were food consumption data [2] and food composition data [24] as described below. We estimated optimal Box-Cox transformations for achieving approximate normality, and checked by visual inspection if a normal approximation was appropriate [19].

Estimating the usual intake distribution allows to calculate not only the mean nutrient intake, but also the percentage of the population at risk of not complying with nutrient requirements. In addition, the contribution of foods to nutrient intakes was given.

The usual nutrient intake distributions were compared with intake limits to estimate the percentage of the population meeting the nutrient requirements using the “cut-point method” (see Background)). For the intake limits of beneficial nutrients we used the Average Nutrient Requirements (ANRs), which is the international harmonized term as proposed by the United Nations University [23], also known as Estimated Average Requirements (EARs) [22]. For a selection of nutrients for which intakes need to be limited, maximal intake limits were used as defined by the FAO/WHO (population nutrient intake goals) [1] and by the Health Council for the Netherlands (for sodium) [25].

### Food Consumption Data

Food consumption data were based on the Dutch National Food Consumption Survey 2003 [2]. This survey was conducted among 750 Dutch young adults (aged 19–30) by trained dieticians using two independent computerized 24-h dietary recalls.

### Food Composition Data and Food Replacement Scenarios

Food composition data from the Dutch food composition database (NEVO, 2006) [24] were evaluated against the Choices criteria [9]. For scenario 1, this food composition database was used as published. For scenarios 2 and 3, all foods that were reported to be consumed by the participants in the survey and that did not comply with the Choices criteria were replaced by similar foods that did comply with the Choices criteria. In a few cases it was not possible to find a replacement food. In these cases, the food was either not replaced (e.g. egg) or occasionally replaced by a better alternative, which still did not comply with the criteria (e.g. chocolate was replaced by sugar free chocolate). In total around 430 foods out of the more than 1600 items in Dutch Food composition database [24] were replaced.

A small number of new foods with the Choices stamp were added to the database and used as replacement foods. Information on the composition of these products was taken from the label. In addition, about 350 foods and their compositions were added to the food composition table. These foods were consumed less frequently in the 2003 survey, and therefore not taken up in the published food composition table. For these foods the sodium, PUFA and MUFA content needed to be estimated based on similar food products of the food composition database. This approach enabled us to estimate the potential (maximum) shift in intakes, while staying as close as possible to the eating habits.

Added sugar is one of the key nutrients with criteria for logo eligibility. However, in the Dutch food composition database, there are only data available on total sugar. For the evaluation of foods against the Choices criteria, estimates for added sugar were used based on other food composition databases [26], [27]. The calculations of the outcome variables was based on the original food composition data [24] and are therefore given for total sugar only. In Table 1 examples of replacements are given.

**Table 1:** Examples of replacements by Choices compliant products from the Dutch Food composition table [24].

Product group	Product example	Replaced by
Carbohydrate sources	Breakfast cereals: muesli, cornflakes	Oat, or wholegrain cereals for porridge
	Bread (various), croissant	Malt bread
	Macaroni	Whole grain macaroni
Fruit & vegetables	Fruit prepared (canned, with syrup, mixed fruit)	Fresh fruit: apple, apricot, pineapple, mixed fruit, cherry, mandarin, pear, peach, applesauce without added sugar
	Processed vegetables (canned, with cream, mixed vegetables)	All processed vegetables were replaced by the same vegetables, prepared (Na-), if not available in food composition table fresh was chosen, if not available raw
	Olives, tomato puree	Not replaced
Dairy products	Full fat/raw milk	Semi-skimmed milk (2%)
	Cream, (crème fraiche, hüttenkäse)	Quark (low fat)
	Hard cheese 48+	Cheese 30+
Spreads and dressings	Margarines, frying fat (various 60/70/80/97% fat), pork fat, butter	Margarine, frying fat, oil (60/70/80/97% fat) with same fat content, meeting SAFA criterion
	Various emulsion based table sauces	Low fat mayonnaise
Fish and meat products	Mackerel (steamed, oil)	Mackerel in water
	Various prepared pork meat (products)	Pork <10 g fat prepared average
	Beef, lamb, horse meat (various, raw)	Beef <5 g fat) average, raw
	Egg, liver	Not replaced
Beverages	Alcoholic drinks	Not replaced
	Soft drinks	Soft drinks light
All other foods	Bread toppings (non-chocolate based)	Jam, no added sugar
Snacks	Cookies, small size pastry	Small muesli bar, portion pack cookies; whole grain biscuit
	Pies, big size pastry^1^	Small muesli bar, portion pack cookies; whole grain biscuit
	Big size chocolate bars^1^	Small muesli bar, portion pack cookies; whole grain biscuit
	Salty snacks (various) and nuts	Mixed nuts
	Big salty snacks^1^ (Dutch: croquet)	Mixed nuts

SAFA: saturated fatty acids.

1 These snacks were not replaced in scenario 4, where only part of the snacks were replaced.

### Energy Correction

For scenario 3, an energy adjustment was applied: Scenario 2 showed a reduction in energy intake with consumption of a Choices compliant diet. It was hypothesised that consumers may compensate for this decrease in energy intake by eating more of a food with a lower energy density (kcal/g). Therefore, when a food (e.g. full fat 48+ cheese: 384 kcal/100 g) was replaced by a food with a lower energy density (in this case: reduced fat 30+ cheese: 279 kcal/100 g), a multiplication factor was applied (in this case: 384/279 = 1.38) so that the total amount of consumed energy was the same as the amount of energy delivered by the food that was replaced. This was done on a product by product basis with exceptions for low calorie soft drinks and some dairy and meat products, because correction would lead to unrealistic amounts being consumed. For these foods, an upper limit was set at 300% for meat and dairy products and 100% for soft drinks.

### Snacks Scenarios

For scenario 2 all non-complying snacks were replaced by healthier alternatives. There were however, very few snacks in the food composition table [24] that were eligible for a healthier choice stamp. To replace all snacks, including apple pies, chocolate bars and other energy dense foods by the few healthier alternatives such as rice wafers and muesli bars seemed unrealistic. Therefore scenario 4 and 5, were calculated, in which snacks were either not replaced or partially replaced. In the partially replaced snack scenario, snacks were only replaced when the healthier alternative was reasonably realistic for e.g. a biscuit or a cookie. High energy apple pies with whipped cream or chocolate bars were not replaced (Table 1). This resulted for this scenario, in the replacement of approximately 40% of the snacks.

### Stratified Analyses

Stratified analyses were performed to explore if the scenarios would have a different effect for subgroups. Intake distributions were stratified for gender (men/women) age in years (<25/≥25), BMI kg/m^2^ (<25/≥25) and education (low/middle/high) and compared to the results of the actual scenario.

## Results

### Changes in Nutrient Intakes

**Figure 2:**
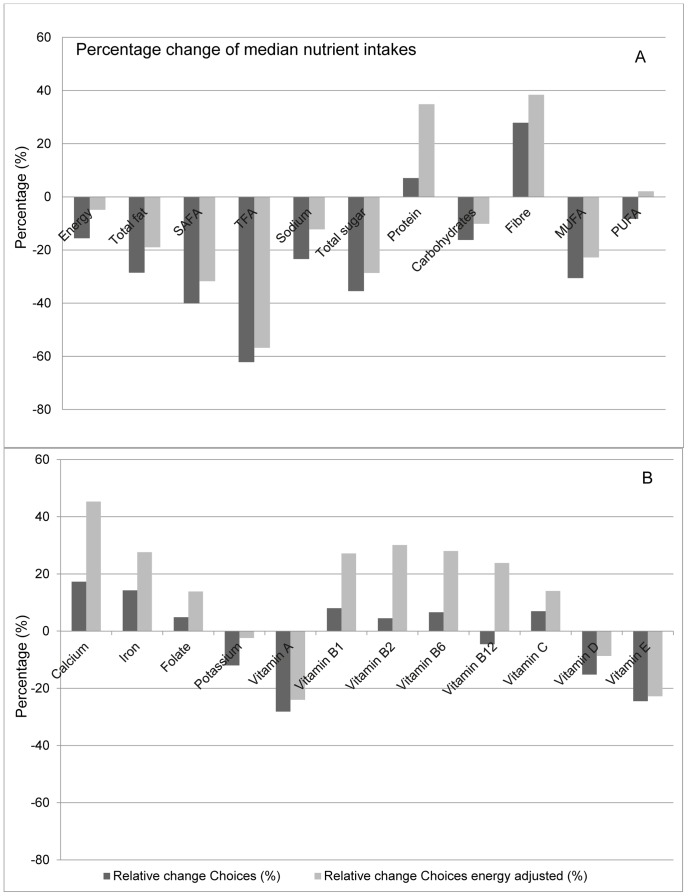
Potential impact on nutrient intakes. Results are expressed as percentage change in median intakes for macronutrients (A) and micronutrients (B) if the Dutch population (aged 19–30) would eat ‘only’ foods that comply with Choices (with and without adjustment for differences in energy density) as compared to the “actual” intake. Assuming a normal distribution, median values approximate average intake levels. SAFA: saturated fatty acids, TFA: trans fatty acids, MUFA: mono unsaturated fatty acids, PUFA: poly unsaturated fatty acids.

Figure 2a shows the percentage change in median nutrient intakes compared to the “actual” intake scenario for energy, fat, SAFA, TFA, sodium, total sugar, fibre and also for protein, total carbohydrates, monounsaturated fatty acids (MUFA) and polyunsaturated fatty acids (PUFA). A reduction was seen for energy intake (−16%), as well as for nutrients with a maximal intake limit (between −23% for sodium and −62% for TFA); but also for total carbohydrates (−16%), MUFA (−31%) and PUFA (−8%). When the data were adjusted for energy intake, these reductions were still present, but reversed for PUFA (+2%).

Figure 2b shows the percentage change in median nutrient intakes for micronutrients compared to the “actual” intake scenario: calcium, iron, folate (natural), and the vitamins A (as retinol equivalents), B_1_, B_2_, B_6_, B_12_ C, D, E. Increases were seen for fibre (28%), calcium (17%), iron (14%) and folate (5%) intake when the Choices scenario was applied. Increases were shown as well for vitamin B_1_ (8%) and B_6_ (7%). All these changes were even larger when the data were adjusted for energy. A decline was observed for potassium (−12%) and vitamin B_12_ (−5%) although the effects were neutralised or even reversed when adjusted for energy (−2%) and (24%) respectively.

For the fat soluble vitamins negative changes were observed (between −28% for vitamin A, −15% for vitamin D and −25% for vitamin E). The decline was proportional compared to the decrease in total fat intake (−29%) and became smaller after adjustment for energy intake.

### Comparison with Dietary Recommendations

The usual intake distributions were compared to nutrient requirements (Population intake goals and ANRs, Table 2 and 3). For this comparison the BBN model was applied: When usual intakes were estimated, variation is reduced for many nutrients, when compared with intakes on a person-day basis calculated directly from the survey data (see Background). This so-called “shrinkage factor” for the nutrients (in the “actual” scenario) ranged from 0.45 to 0.71. To illustrate this, for example, without this correction, the percentage of the population with insufficient vitamin B_12_ intakes in the “actual” scenario (shrinkage factor 0.53) would have been estimated erroneously as 24.3% instead of 5.9% (Table 3).

**Table 2:** Percentage of the population with intakes that do not comply with the population nutrient intake goals for different nutrients in Dutch adults aged 19–30 year.

Nutrient	Population nutrient intake goals^1^	Percentage not complying with (higher than) population nutrient intake goals		
	Maximum	Actual	Choices	Choices energy adjusted
SAFA	10 en% (22 g/d)	87.7	36.0	52.1
TFA	1 en% (2.2 g/d)	51.2	0.1	0.4
Sodium	2400 mg/d	80.6	39.8	62.5
Total sugar	15 en% (75 g/d)	94.6	71.1	79.7

SAFA: saturated fatty acids, TFA: trans fatty acids.

1 Population nutrient intake goals are published as percentage of energy [1]; these recommendations were translated to g/d based on a 2000 kcal diet; for sugar this resulted in 75 g of total sugar per day, assuming that total sugar intakes are made up of 2/3 added/free sugar [15].

**Table 3:** Percentage of the population with intakes that do not comply with the Average Nutrient Requirements (ANRs) for different nutrients in Dutch adults aged 19–30 year.

Nutrient	Average Nutrient Requirements (ANR)^1^	Percentage^5^ not complying with (lower than) Average Nutrient Requirements		
	Minimum	Actual	Choices	Choices energy adjusted
Iron	7 mg/d^2^	6.8	2.8	1.0
Folate	200 μg^/^d	67.0	59.5	47.8
Vitamin A	575 μg/d^3^	13.5	33.9	27.7
Vitamin B_1_	0.8 mg/d	8.0	4.1	1.4
Vitamin B_2_	1.0 mg/d^4^	12.5	10.1	4.2
Vitamin B_6_	1.1 mg/d	6.4	3.5	1.0
Vitamin B_12_	2.0 μg/d	5.9	5.1	0.7
Vitamin C	67.5 mg/d	26.6	24.8	21.2
Vitamin E	12 mg/d	64.5	90.5	89.2

1 Average Nutrient Requirements (ANR), also known as Estimated Average Requirements (EAR) are defined as level of intake sufficient to meet the requirement for half of the healthy individuals in a particular life stage and gender group. The ANRs are the same as used in the most recent Dutch National food consumption survey 2007–2010 [3].

2 For iron: average for male, 6 g/d and female, 8.1 g/d;

3 For vitamin A: average for male, 620 µg/d and female, 530 µg/d;

4 For vitamin B2: average for male, 1.1 mg/d and female, 0.8 mg/d.

5 Percentage of the population with nutrient intakes insufficient for their personal requirements. In other words: the percentage at risk for inadequacy.

With the “actual” scenario, intake limits for the key nutrients SAFA, TFA, sodium and total sugar were not reached by 88, 51, 81 and 95% of the population respectively. After replacing non-complying products with products complying with Choices criteria, the proportion of the population not meeting recommended intake limits reduced to 36, 0.1, 40, and 71% respectively. After adjustments for energy, the percentage that did not comply with the criteria for SAFA, TFA, sodium and total sugar increased (Table 2).

The results for beneficial nutrients varied. After replacement with products complying with the Choices criteria, the percentage of the population at risk of intakes lower than the Average Nutrient Requirements (ANRs) decreased for iron, most of the B-vitamins and vitamin C. However, the opposite was shown for the fat soluble vitamins (A, E). The percentages of the population at risk of lower intakes than the ANRs for vitamin A and vitamin E increased to 34% and 91%, respectively (Table 3). For vitamin D, calcium, fibre and potassium no ANR was established.

**Figure 3:**
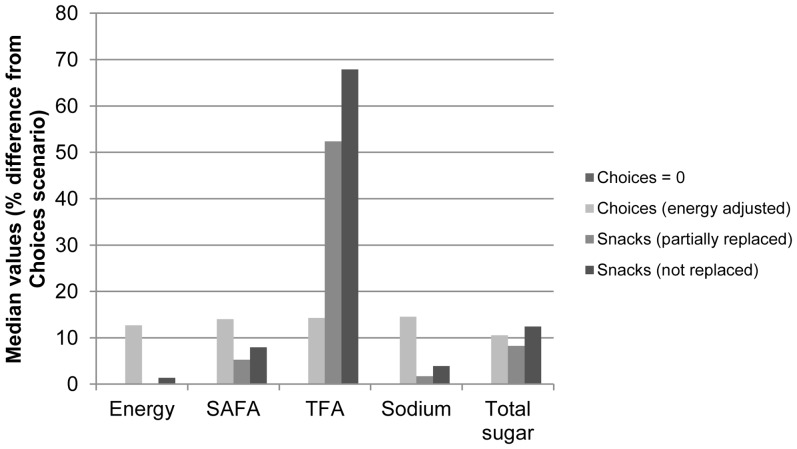
Potential impact of snacks replacement (partially or not) on intakes of energy, SAFA, TFA, sodium and total sugar. Results are expressed as percentage difference of median nutrient intakes for the different scenarios as compared to the Choices scenario: If the Dutch population (aged 19–30) would eat ‘only’ foods that comply with Choices (set at  = 0), with adjustment for differences in energy density (Choices energy adjusted), with partial or with no replacements of snacks (Snacks, partially or not replaced). Assuming a normal distribution, median values approximate average intake levels. SAFA: saturated fatty acids, TFA: trans fatty acids.

**Figure 4:**
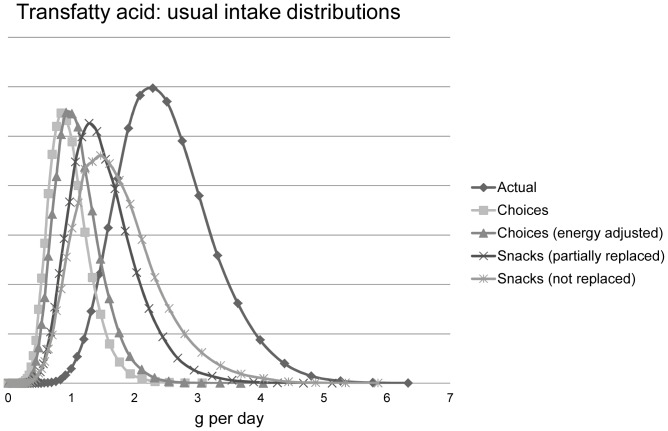
Usual intake distributions for trans fatty acids (TFA) for all five scenarios. “Actual” TFA intake is compared to the Choices scenario: if the Dutch population (aged 19–30) would eat ‘only’ foods that comply with Choices; with and without adjustment for differences in energy density (Choices and Choices, energy adjusted); with partial or with no replacements of snacks (Snacks, partially or not replaced). Maximal intake limit for TFA is 2.2 g/d (1 en%, 2000 kcal).

Table 4 shows the intake distributions for energy SAFA, TFA, sodium, and total sugar for the five different scenarios, including the different snack-scenarios. When evaluating the median values (Figure 3), the snacks scenarios indicated that not replacing snacks with Choices compliant alternatives resulted in substantially higher intakes for sugar (12%) and SAFA (8%) and especially TFA (68%), indicating snacks being important sources of TFA and also, but to a lesser extent, sugar and SAFA. For energy and sodium, these effects were much smaller (1 and 4%, respectively). The intake distributions became narrower for the Choices scenario and the snack scenarios, as can be seen by lower inter-quartile ranges (IQRs). This indicates less variability and shorter tails of the distributions (Table 4, Figure 4). This is also illustrated for TFA in Figure 4 where the distributions are shown for all five scenarios.

**Table 4:** Percentiles of nutrient intakes according to different scenarios.

Nutrients to limit		Percentiles					
	Scenarios	p2.5	p25	p50	p75	P97.5	IQR
Energy (kcal)	*Actual intake*	1289	1900	2274	2699	3630	799
	*Choices*	1079	1594	1921	2284	3083	690
	*Choices energy adjusted*	1222	1802	2164	2571	3450	769
	*Snacks (partially replaced)*	1068	1597	1924	2290	3088	693
	*Snacks (not replaced)*	1083	1616	1947	2316	3130	699
SAFA (g)	*Actual intake*	16.1	26.2	32.8	40.1	56.7	13.9
	*Choices*	9.4	15.6	19.6	24.3	34.8	8.7
	*Choices energy adjusted*	10.8	17.8	22.4	27.6	39.4	9.8
	*Snacks (partially replaced)*	9.9	16.4	20.7	25.6	36.6	9.1
	*Snacks (not replaced)*	10.1	16.9	21.2	26.2	37.4	9.3
TFA (g)	*Actual intake*	1.2	1.8	2.2	2.7	3.9	0.9
	*Choices*	0.4	0.7	0.8	1.1	1.6	0.4
	*Choices energy adjusted*	0.5	0.8	1.0	1.2	1.8	0.4
	*Snacks (partially replaced)*	0.6	1.0	1.3	1.6	2.5	0.6
	*Snacks (not replaced)*	0.6	1.1	1.4	1.8	3.0	0.8
Sodium (mg)	*Actual intake*	1804	2514	2949	3426	4474	912
	*Choices*	1385	1926	2261	2637	3469	710
	*Choices energy adjusted*	1575	2199	2590	3027	3988	829
	*Snacks (partially replaced)*	1390	1956	2300	2685	3540	728
	*Snacks (not replaced)*	1431	1995	2349	2743	3628	748
Total sugar (g)	*Actual intake*	63.6	110.8	142.0	177.6	261.0	66.8
	*Choices*	42.6	71.8	91.7	115.3	171.3	43.5
	*Choices energy adjusted*	47.0	79.6	101.3	126.5	186.6	46.9
	*Snacks (partially replaced)*	47.3	78.6	99.3	123.5	179.4	44.9
	*Snacks (not replaced)*	48.8	81.2	103.1	128.5	187.7	47.3

SAFA: saturated fatty acids, TFA: trans fatty acids, IQR: inter quartile range (P75 - P25).

In Table 5 the foods that contribute to the intake of the SAFA and vitamin A are given. For SAFA, dairy products (milk, cheese), meat and fats are major contributors in the “actual” scenario. For the Choices scenarios, major contributors to SAFA intake are dairy products and snacks.

**Table 5:** Top 10 foods according to the “Actual” and “Choices” scenarios for the intake of saturated fatty acids and vitamin A given as percentage of total intakes.

	Actual		Choices	
	Food	Percentage	Food	Percentage
**Saturated fatty acids**	Hard cheese (48+ Gouda)	13.2	Cheese 30+	14.5
	Semi-skimmed milk (2%)	4.8	Semi-skimmed milk (2%)	8.5
	Crisps	3.3	Granola bar	6.7
	Butter (not salted)	2.5	Hot Chocolate (without sugar)	6.6
	Minced meat	2.35	Margarine (80% fat)	5.6
	Margarine (80% fat)	2.05	Mixed nuts	3.7
	Margarine (40% fat)	1.84	Margarine (40% fat)	3.5
	Sausage (smoked)	1.29	Wheat bread	2.9
	French fries	1.16	Cookies (Kids)	2.6
	Coffee creamer	1.08	French Cheese (Camembert 45+)	2.2
	**Sum (top 10 foods)**	**33.5**		**56.7**
**Vitamin A**	Liver-based meat product	6.9	Carrots (boiled)	17.0
	Carrots (boiled)	6.2	Margarine (40% fat)	14.1
	Liver-based meat product (spreadable)	5.1	Margarine (80% fat)	10.0
	Liver-based meat product (spreadable)	4.8	Red sauce	7.1
	Margarine (40% fat)	4.7	Semi-skimmed milk (2%)	5.9
	Hard cheese (Gouda 48+)	3.9	Multivitamin nectar	5.8
	Curly kale (boiled)	3.0	Cheese 30+	4.1
	Multivitamin nectar	2.8	Pate (< less fat)	3.2
	Semi-skimmed milk (2%)	2.7	Cooking fat 97% fat	2.6
	Liver-based meat product	2.6	Mandarin	2.5
	**Sum (top 10 foods)**	**42.7**		**72.5**

For vitamin A, intakes were substantially lower with the Choices scenarios. Table 5 shows that when high-fat, liver-based meat products, were in the Choices scenarios replaced by meat sources with lower vitamin A content, this resulted in a larger contribution of carrots, and fats to overall vitamin A intake. Carrots are in general 10–20 times less rich in vitamin A (or carotene) as compared to liver based meat products [24]. In the Choices scenarios, foods that contribute to the intake of vitamin E shift from mayonnaise as a main contributor towards nut-based products. In addition, fats remained an important source of vitamin E. In all scenarios, vitamin D came from fat, meat and fish products (data not shown).

The results of the analysis stratified for gender showed only marginal differences. In general, women showed slightly larger changes than men −65.0% vs. −60.0% for TFA, respectively, when the Choices criteria were applied, except for most vitamins were the effects in men were slightly larger (vitamin E, −20.9% women vs. −27.9% men). A close look at the BMI groups showed that the values of the usual intakes of the high BMI group were consequently lower than the low BMI group (energy 2137 vs. 2343 kcal). However, despite these differences in intakes, the Choices scenario and the Choices energy adjusted scenario had comparable effects for different BMI groups. Stratification for education and age resulted only in minor differences in impact between the groups (data not shown).

## Discussion

In this nutrient intake modelling study we used the MCRA model to investigate the potential impact of the Choices Programme on nutrient intakes. In general the nutrient intake distributions based on the 2003 Dutch food consumption survey shifted into the direction of the recommendations [1] when non-complying foods were replaced with Choices compliant products.

Using nutrient intake modelling, we can evaluate whether a nutrient profiling system is able to improve the nutritional quality of consumers’ diets. The calculation of intake distributions enabled us to calculate the fraction of the population that has a long-term average nutrient intake which is above or below nutrient requirements (FAO/WHO population nutrient intake goals or ANR). This approach allows a quantitative look into effects of specific reformulations, on intakes from specific food groups, such as snacks, possible differences in intakes related to population characteristics (such as BMI, gender and education). The model calculated the contribution of specific foods to nutrient intakes (Table 5) and has the possibility to include market share information, as done by Temme et al. [28].

The results for the nutrients saturated fatty acids (SAFA), trans fatty acids (TFA), sodium, sugar, energy and fibre, for which criteria are defined for logo eligibility in the Choices Programme, showed a shift in intake distributions in a beneficial direction when the population would consume a diet conform the Choices criteria. With regard to total sugar intakes, the results were less strong. While there was an improvement in the percentage of the population meeting the recommended intake limit for sugar, a substantial proportion of the population would still consume more than is recommended when non-compliant foods were replaced with Choices compliant foods. One reason for this could be that the Choices criteria for added sugar are not strict enough. However evidence for an optimal level of added sugar intake does not allow firm conclusions [1].

Regarding the macronutrients, protein intake increased probably due to the increased consumption of low-fat, protein-rich, animal based products. The replacement of non-complying foods with Choices compliant products decreased the intakes of carbohydrates and unsaturated fatty acids. Regarding the minerals and micronutrients, potassium intakes were reduced. In addition, the percentage of the population that had estimated intakes below the Average Nutrient Requirements (ANR) was higher for the fat-soluble vitamins (A and E). This unintentional effect could be related to the reduction in total fat intakes and the replacement of foods such as high-fat, liver-based meat products that are specifically high in, for example, vitamin A (Table 5). Also, high fat mayonnaise, which is non-compliant because of energy density, was a good source of vitamin E. For vitamin D, it was less clear which food replacement caused the reduction. Intakes of micronutrients like calcium, iron and the water soluble vitamins were higher with the Choices scenarios, resulting in a reduction of the percentage of the population at risk for inadequacies. It must be noted that the ANR represents the average daily nutrient intake level estimated to meet the requirement of half of the healthy individuals in a defined population [2], [22]. De Lauzon *et al*. validated and justified the ANR as a cut-point for the prevalence of nutrient inadequacy at a population level [29]. For nutrients for which no ANR was defined such as vitamin D, calcium, fibre and potassium, this cannot be applied. We compared the percentages of the population at risk for inadequacies in the “actual” scenario with those in the same age group in the latest Dutch survey [3]. For iron, vitamin B_1_, B_6_, B_12_ and C, the numbers were comparable. We found a higher percentage of the population at risk for low intakes of folate, vitamin B_2_ and E, while for vitamin A this percentage was lower. Some of these differences (folate and vitamin E) were larger than expected based on anticipated differences based on weighing factors or effect of time (2003 vs. 2010) between two surveys of the same age group in the same population. Therefore these absolute percentages of the population at risk for inadequacies need to be interpreted with caution. Alternatively, the relatively low intakes of some of the beneficial nutrients might also be related to the fact that food composition data is incomplete for some of the micronutrients, leading to underestimated intakes and higher estimated risks for inadequacies.

A way to overcome the unintentional negative side-effects on mineral and micronutrient intakes could be the inclusion of criteria for these nutrients in the Choices Programme as is done in other nutrient profiling systems [10], [30]. This would, however, complicate implementation of such a nutrient profiling system, because it is necessary to collect or analyse these data, for the evaluation of foods for stamp eligibility. Recently, effects on product group-specific (micro)nutrient intakes of different nutrient profile systems have been estimated [31]. The authors illustrated that a simultaneous decrease of estimated vitamin D and calcium intakes with a reduction in SAFA and sodium from dairy products, is affected by the type of nutrient profiling system and the inclusion of criteria for beneficial nutrients [31].

To compensate for the difference in energy density between the original and the replacement foods a multiplication factor was applied. The energy adjusted scenario can be seen as the “worst case scenario”, assuming consumers may compensate for the decrease in energy intake when switching to Choices compliant products. Nevertheless, results still showed a substantial shift in intake distributions into a more favourable direction. Stratified analysis for gender, BMI, education and age resulted only in marginal differences between the subgroups. This means that Choices had comparable effects for all subgroups in terms of potential impact on nutrient intakes.

In addition to the studies mentioned above [29], [31], also others reported on food replacement scenarios, mostly with a focus on SAFA only [32]–[34]. Schickenberg *et al* [32] replaced up to three products from three different product groups with low-SAFA alternatives, resulting in a mean reduction of 13.4 g SAFA. Lloyd Williams *et al* [35] replaced only one snack with a healthier alternative resulting in a smaller reduction of 4.4 g SAFA per day (−120 kcal). In the present study the median SAFA reduction for a total diet was larger (13.2 g, Table 3). However, we estimated a smaller contribution of snacks to median SAFA intake of 1.5 g (Table 4). An explanation for the different findings in these studies [32], [35] can be that in our study the replacements were chosen to stay as close as possible to the normal situation. For example, to define a ‘realistic’ replacement, we replaced full fat cheese (Gouda 48+) by cheese 30+ instead of cheese 10+ as was done by Schickenberg *et al* [32]. The potential effect of Choices on the general Dutch population (18–70 y) was studied by Vyth et al [33] who showed a reduction of average SAFA intake between 10 to 15 en% and for TFA 0.6 to 1.0 en%. These numbers related to a broader age-range of the Dutch population (18–70 y) are in the same range as our values (SAFA from 9 to 13 en% and TFA from 0.9 to 0.4 en%) for a population aged 19–30 y. In addition, results for SAFA, TFA and sodium intakes were also comparable as estimated in a study using a different methodology (Daily Menu Method) for measuring potential effects of the Choices Programme on nutrient intakes [34]. Estimated intakes of (added) sugar were less reduced in the present study as compared to our earlier work, which was more subjective and lacked good added sugar data.

However, the present study may also have some limitations. Despite the fact that replacements were chosen on a product by product basis, dependent on the decisions of two nutritional experts, this may however still be susceptible to some subjectivity and bias. Also product acceptability is not taken into consideration. Consumers might prefer other alternatives. For sweet and salty snacks there were few Choices compliant replacements, thus the same replacement food has been used for a large number of snacks (Table 1), leading to unrealistic high consumption of a few snack products. Furthermore, snacks are usually eaten for indulgence; therefore it is unrealistic to assume that consumers will replace all snacks with one and the same healthier alternative.

Moreover, the use of generic food composition data such as NEVO [24] could be criticized because nutrient profiling systems are intended to be implemented on real food products. Many branded products are not included, meaning that several products are averaged to one nutrient composition, which may have influenced individual intakes. New products are not in the food composition table. Nutrient data may not be correctly measured or calculated. But, these generic food composition databases are the best open source of nutritional information of a wide range of foods and are used in surveys. Alternatives are food composition data from labels, especially useful for countries where labelling is obligatory. The role of food composition data in the benchmarking and evaluations of foods highlights the need for good up-to-date, quality data on nutrient composition of foods in the supermarket. This is a challenge for the fast changing global food supply, which is the reality in the current market.

Another limitation is the fact that survey data are known to be associated with underreporting of intake especially in subjects with a higher BMI [36]. Therefore, results indicating the percentage of the population that complied with the nutrient requirements have to be interpreted with caution since it may be lower (for nutrients to limit) or higher (for beneficial nutrients). However, stratified analyses indicated that BMI was not likely a disturbing factor in the present study.

Despite mentioned limitations, this study offers opportunities for future research which may include quantifying the beneficial effects of these same modest changes on health outcomes for example chronic diseases and the disease burden. For example, Lloyd-Williams [35] showed that approximately 6000 cardiovascular deaths could be prevented annually in the UK by reducing cholesterol levels with 0.054 mmol/L and salt intake with 0.5 g on population level. Vyth et al. estimated slight positive potential effects on cholesterol levels [33].

In conclusion, data from this study in a Dutch young adult population shows the potential beneficial effects of Choices on nutrient intakes. There are, however concerns for some beneficial nutrients. Especially estimated fat-based beneficial nutrient intakes were unintentionally reduced with replacement of high-fat foods by alternatives with a better fat quality or lower energy content. It is recommended to study this effect in more detail. For the nutrients used in the Choices benchmarks, intakes shift substantially in a beneficial direction when people consume Choices compliant foods. By choosing healthier options in each product category, consumers could have substantially healthier diets that are more in line with the WHO recommendations. These changes could potentially have significant impact on public health in terms of chronic diseases. The results of the simulation study are promising, although field studies that monitor nutrient intakes are needed to confirm the feasibility and impact of the proposed strategy in real life.
